# The pattern of congenital heart defects arising from reduced Tbx5 expression is altered in a Down syndrome mouse model

**DOI:** 10.1186/s12861-015-0080-y

**Published:** 2015-07-25

**Authors:** Renita C. Polk, Peter Gergics, Jeffrey D. Steimle, Huiqing Li, Ivan P. Moskowitz, Sally A. Camper, Roger H. Reeves

**Affiliations:** Department of Physiology at Johns Hopkins, Biophysics 201, 725 N. Wolfe St., Baltimore, MD 21205 USA; McKusick Nathans Institute for Genetic Medicine, School of Medicine, Johns Hopkins University, Baltimore, MD USA; Departments of Pediatrics, Pathology and Human Genetics, University of Chicago, Chicago, IL USA; Department of Human Genetics, School of Medicine, University of Michigan, Ann Arbor, MI USA

**Keywords:** Heart development, Congenital heart defect, Down syndrome, Trisomy

## Abstract

**Background:**

Nearly half of all individuals with Down Syndrome (DS) have some type of congenital heart defect (CHD), suggesting that DS sensitizes to CHD but does not cause it. We used a common mouse model of DS, the Ts65Dn mouse, to study the contribution of *Tbx5,* a known modifier of CHD, to heart defects on a trisomic backgroun. Mice that were heterozygous for a *Tbx5* null allele were crossed with Ts65Dn mice. Thoraxes of progeny were fixed in 10% formalin, embedded in paraffin, and sectioned for analysis of CHD. Gene expression in embryonic hearts was examined by quantitative PCR and in situ hybridization. A TBX5 DNA binding site was verified by luciferase assays.

**Methods:**

Mice that were heterozygous for a Tbx5 null allele were crossed with Ts65Dn mice. Thoraxes of progeny were fixed in 10 % formalin, embedded in paraffin, and sectioned for analysis of CHD. Gene expression in embryonic hearts was examined by quantitative PCR and in situ hybridization. A TBX5 DNA binding site was verified by luciferase assays.

**Results:**

We crossed mice that were heterozygous for a *Tbx5* null allele with Ts65Dn mice. Mice that were trisomic and carried the *Tbx5* mutation (Ts65Dn;*Tbx5*^*+/−*^) had a significantly increased incidence of overriding aorta compared to their euploid littermates. Ts65Dn;*Tbx5*^*+/−*^ mice also showed reduced expression of *Pitx2,* a molecular marker for the left atrium. Transcript levels of the trisomic *Adamts1* gene were decreased in *Tbx5*^*+/−*^ mice compared to their euploid littermates. Evidence of a valid binding site for TBX5 upstream of the trisomic *Adamts1* locus was also shown.

**Conclusion:**

Haploinsufficiency of *Tbx5* and trisomy affects alignment of the aorta and this effect may stem from deviations from normal left-right patterning in the heart. We have unveiled a previously unknown interaction between the *Tbx5* gene and trisomy, suggesting a connection between *Tbx5* and trisomic genes important during heart development.

**Electronic supplementary material:**

The online version of this article (doi:10.1186/s12861-015-0080-y) contains supplementary material, which is available to authorized users.

## Background

Congenital heart defects (CHD) comprise the most common congenital anomaly in live births [1]. There is an especially high incidence of CHD in Down syndrome (DS) as 40 – 50 % of individuals with trisomy for human chromosome 21 (Hsa21) are affected [2–7]. In particular, atrioventricular septal defect (AVSD) occurs in about 20 % of people with DS, a frequency about 2000-fold higher than in the population at large [8]. Since half of those with DS have a normal heart, additional genetic and environmental factors must interact with DS to cause CHD. Thus, dosage effects of Hsa21 genes comprise a complex modifier that, in conjunction with other risk factors such as single-gene variants, alters outcomes of heart development. Trisomy can be thought of as a genetically sensitizing condition that de-stabilizes normal heart development, unveiling roles of disomic modifiers of CHD [8, 9]. Attempts to identify genes predisposing to CHD in DS have understandably focused on Hsa21, while there has been little consideration of disomic modifiers that may contribute to this increased risk.

We consider the role of *Tbx5,* a known contributor to heart development, as a modifier and assess its interaction with trisomy*. Tbx5*, a transcription factor, has well-described effects in cardiac and limb development [10, 11]. Mutations in this gene are associated with Holt-Oram syndrome and about 85 % of affected individuals have a structural heart defect and/or abnormalities in the cardiac conduction system. Holt-Oram patients most often present with atrial septal defects (ASDs) and ventricular septal defects (VSDs) [12]. *Tbx5* has a well-studied role in the morphogenesis of the four heart chambers. It is expressed in the left ventricle (LV) and both atria during chamber maturation and septation [13]. Ectopic expression in the right ventricle (RV), or deletion of *Tbx5* in the left ventricles (LV) of mice suppresses formation of the ventricular septum, resulting in formation of a single ventricle [14]. Its importance in heart development suggests that variants affecting *Tbx5* expression might affect heart development on the “sensitized” trisomic background.

Animal models provide important information for understanding the pathogenesis of CHD and the molecular mechanisms that give rise to these conditions. Orthologs of many genes on Hsa21 are found on mouse chromosome 16 (Mmu16), with smaller subsets on Mmu10 and Mmu17 [15]. The most widely studied DS mouse model, Ts65Dn, is trisomic for a segment of Mmu16 containing about half of the mouse genes orthologous to Hsa21 [16]. The freely segregating extra chromosome carrying these genes also contains genes from Mmu17 that are not conserved with Hsa21, thus this is not an exact model [17, 18]. Ts65Dn mice display a number of the features of DS, including cardiac abnormalities, although these occur at a lower frequency than in people with DS [9, 19]. Our lab has previously identified the *Creld1* and *Hey2* genes as disomic modifiers of septal development on this trisomic background [9]. Haploinsufficiency for either of these disomic modifiers alone did not affect heart development, but on a trisomic background the frequency of maldevelopment was increased significantly. The Ts65Dn model thus sensitizes heart development to other genetic perturbations. We used the Ts65Dn mouse model here to examine the role of *Tbx5* in heart development.

A mouse model with a null allele for *Tbx5* has been described [20]. Homozygous null *Tbx5*^−/−^ embryos (Black Swiss/SvJ background) die by embryonic day 10.5 (E10.5) and lack cardiac looping and endocardial cushion formation, among other severe defects [20]. The viability of and defects observed in *Tbx5*^*+/−*^ mice are greatly influenced by genetic background. Bruneau *et al.* report a 10 % frequency of the *Tbx5* null allele on a 129SvEv/129SvJ background at birth and 28 % on a Black Swiss/129SvJ background, instead of the expected Mendelian ratio of 50 %. Deviation from the expected frequency indicates that prenatal loss has occurred; the different frequencies in different mouse strains suggest that genetic background contributes to the penetrance and expressivity of heart phenotypes in this situation [20]. Therefore, the effects of *Tbx5* dosage are susceptible to additional genetic modifiers. The molecular mechanisms by which *Tbx5* influences heart development are incompletely described and possible interactions between *Tbx5* and genes on Hsa21 are unknown. We hypothesize that *Tbx5* acts as a genetic modifier to alter CHD in Ts65Dn mice. Here we provide evidence of an interaction between *Tbx5* and trisomy and the effects of that interaction on trisomic gene expression and left-right patterning of the heart.

## Results

### Viability of *Tbx5*^*+/−*^ mice is dependent on genetic background

Crosses between B6.*Tbx5*^+/−^ male mice and B6C3.Ts65Dn females were established to examine the role of *Tbx5* as a modifier of CHD. Genetic background of the *Tbx5* mice affected viability (Table 1 and [20]). At birth, *Tbx5* genotypes appeared at Mendelian ratios on a B6 x C3H (75 % B6, 25 % C3H) background (Table 2), but by weaning, the frequency of the *Tbx5*^*+/−*^ genotype was 21 % rather than the expected 50 %. On a B6 background, the frequency of the *Tbx5*^*+/−*^ genotype at weaning was 11 %. Thus, genetic factors appear to contribute to perinatal lethality associated with *Tbx5*^*+/−*^.

**Table 1:** Strain dependent viability of euploid *Tbx5* ^*+/−*^ mice at weaning

Genetic background	Frequency of *Tbx5* ^*+/−*^ genotype
*C57BL/6J*	11 % (*n* = 591)
*B6;C3H (75 % B6, 25 % C3H)*	21 % (*n* = 131)
129SvEv/129SvJ	10 %^a^
Black Swiss/SvJ	28 %^a^

^a^[20]

**Table 2:** Ratios of all genotypes in a Ts65Dn x *Tbx5* ^*+/−*^ mating on a B6;C3H (75 % B6, 25 % C3H background at P0

Genotype	Number (%), *n* = 180
*Tbx5* ^*+/+*^	52 (28.9 %)
*Tbx5* ^*+/−*^	51 (28.3 %)
Ts65Dn;*Tbx5* ^*+/+*^	46 (25.6 %)
Ts65Dn;*Tbx5* ^*+/−*^	31 (17.2 %)

**Fig. 1:**
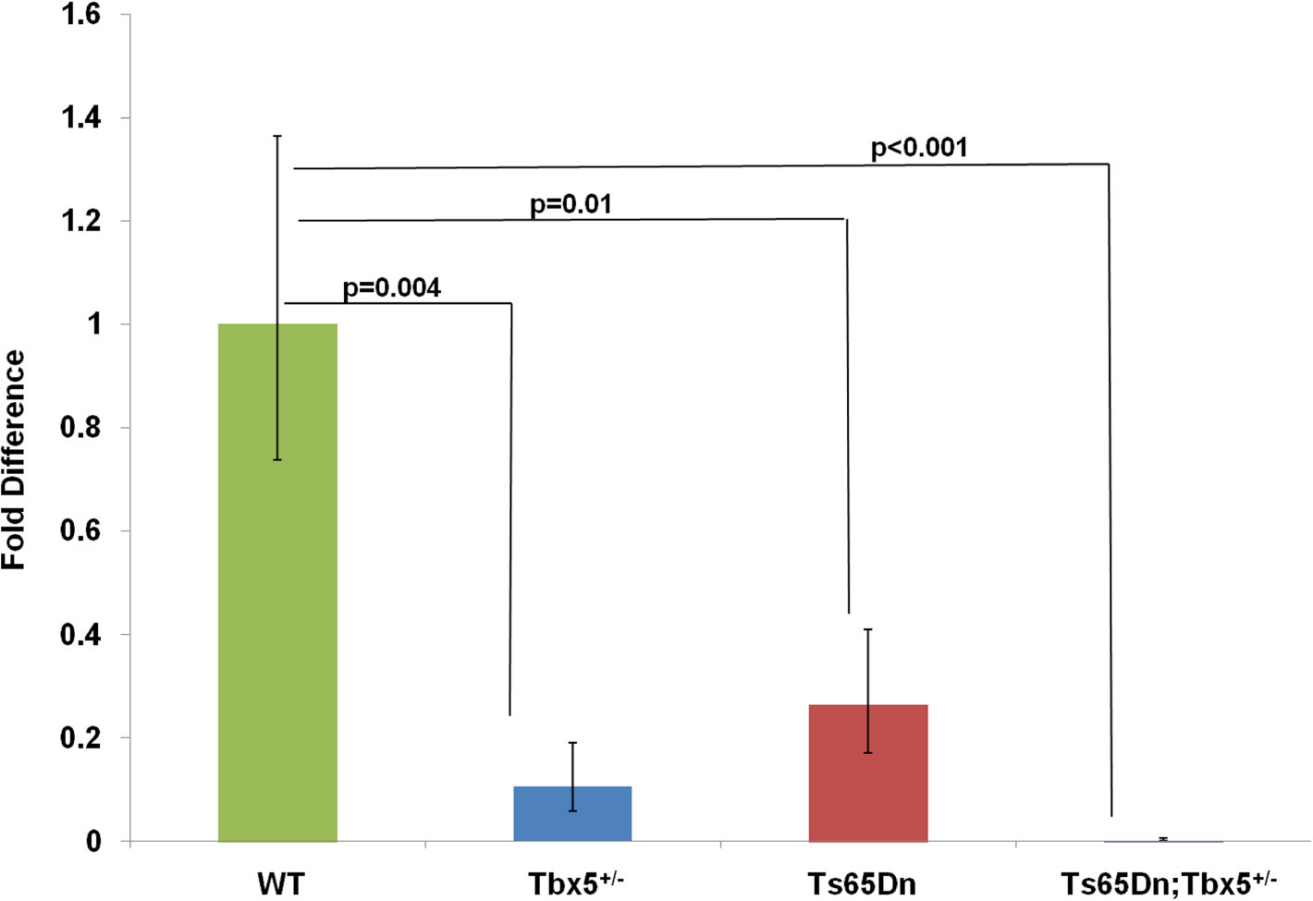
Quantitative PCR of *Tbx5* mRNA levels in pooled E11.5 hearts. mRNA expression was reduced to almost 10 % of wild type levels in *Tbx5* ^*+/−*^ mice (p = 0.004). Note that previous studies found expression levels reduced to 20 % of wild type levels in E8.5 *Tbx5* ^*+/−*^ embryos on a mixed Black Swiss/129 background [21]. We found expression was also reduced to 30 % of wild type levels in Ts65Dn mice (p = 0.01), and 2 % of wild type levels in Ts65Dn;*Tbx5* ^*+/−*^ hearts (p < 0.001). Error bars are standard deviation from the mean

We examined *Tbx*5 expression in hearts at E11.5 and confirmed the expected down-regulation in *Tbx5* heterozygous null mice (Fig. 1). The original characterization of these mice demonstrated Tbx5 RNA levels substantially less than 50 % at E8.5 [21], and the very low expression seen here at E11.5 reflects that difference, as well. Surprisingly, *Tbx5* mRNA was also reduced in Ts65Dn. There may be an additive effect between the two conditions, as the measured levels of Tbx5 mRNA were lower still in Ts65Dn;*Tbx5*^*+/−*^ mice.

### Trisomy affects patterns of CHD in *Tbx5*^*+/−*^ mice

Progeny of the Ts65Dn x *Tbx5*^+/−^ crosses were collected within hours of birth, prepared for histology and assessed for the presence of CHD. All animals examined for CHD were on the B6 x C3H (75 % B6, 25 % C3H) background. There is some loss of Ts65Dn fetuses during late gestation and the observed frequency of 42 % trisomic pups at P0 (Table 2) was in the expected range [22, 23]. *Tbx5*^+/−^ mice were recovered at Mendelian ratios within hours of birth. CHD is highly penetrant in *Tbx5*^+/−^ mice and we saw only a slight overall increase in the percentage of heart defects when the null allele occurred on a trisomic background (Table 3 and Additional file 1: Table S1). However, the pattern of effects was altered significantly by trisomy.

**Table 3:** CHDs in *Tbx5* ^*+/−*^ trisomic and euploid mice^a^

Genotype	ASD	VSD only	Overriding Aorta^b^	Gerbode’s defect	AVSD^c^	No defect	Total mice
*Tbx5* ^*+/−*^	11 (27.5 %)	10 (25 %)	7 (17.5 %)	4 (10 %)	2 (5 %)	13 (32.5 %)	40
Ts65Dn; *Tbx5* ^*+/−*^	12 (38.7 %)	6 (19.4 %)	18 (58.1 %)	6 (19.4 %)	6 (19.4 %)	5 (16 %)	31

^a^Several animals had more than one defect. (see Additional file 1: Table S1)

^b^There is a significant difference between euploid and trisomic Tbx5 heterozygous mice in the occurrence of overriding aorta (p = 0.0004)

^c^The difference between euploid and trisomic Tbx5 heterozygous mice in AVSD is approaching significance (p = 0.07)

**Fig. 2:**
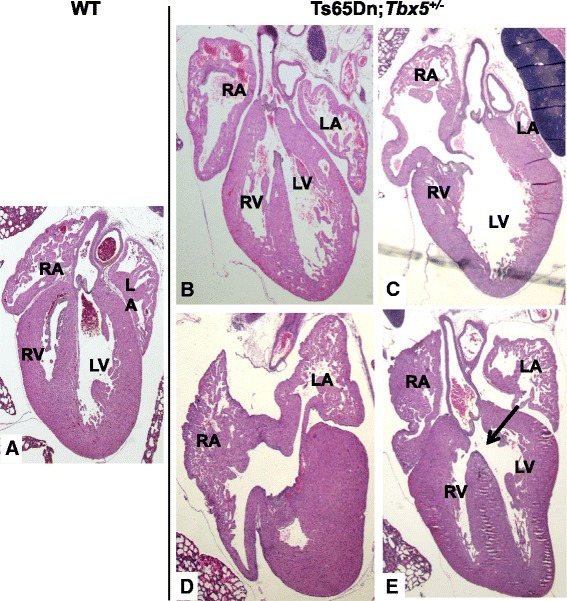
CHDs seen in newborn *Tbx5* ^+/−^ trisomic and euploid mice. Coronal histological sections of wild type (**a**) and Ts65Dn;*Tbx5* ^+/−^ (**b–e**) newborn mice stained with H&E. Overriding aorta (**b**), Gerbode’s defect (**c**), ASDs (**d**), and VSDs (**e**) were seen in the examined animals. RA/LA: right/left atrium, RV/LV: right/left ventricle

We observed overriding aorta (OA) in ~58 % of Ts65Dn;Tbx5^+/−^ mice but only ~18 % of *Tbx5*^+/−^, a significant difference (Table 3, p = 0.0004). OA consists of a VSD and an improperly positioned aorta directly over the VSD (Fig. 2b). The penetrance of AVSD was somewhat elevated in Ts65Dn;Tbx5^+/−^ mice, with ~19 % affected by AVSD vs. 5 % of *Tbx5*^+/−^ mice, although it did not reach formal statistical significance (p = 0.07). ASD and Gerbode’s defect, an abnormal communication between the right atrium and left ventricle (Fig. 2c,d), were also seen more frequently in Ts65Dn;Tbx5^+/−^ but did not reach statistical significance. We performed CT-based Virtual Histology™ on six Ts65Dn;Tbx5^+/−^ mice (Numira Biosciences, Salt Lake City, UT; see Methods). Three of these animals were affected, with an AVSD, ASD and VSD, respectively (see Additional file 2: video 1, Additional file 3: video 2 and Additional file 4: video 3).

### Candidate trisomic gene interaction with *Tbx5*

The simplest explanation for the increased incidence of overriding aorta in Ts65Dn;*Tbx5*^*+/−*^ mice is an interaction between *Tbx5* and a trisomic gene(s) that is regulated by this transcription factor. We considered 109 genes that are trisomic in Ts65Dn mice [24] and generated a list of candidate trisomic genes based on expression patterns (Additional file 5: Figure S1). Sixty-four of these genes are expressed in the heart during development and 43 of them contained regions that associated with TBX5 in a ChIP study [25]. However, examination of the genomic sequences of these 43 revealed a T-box binding element (TBE) in only six (*Adamts1, Dyrk1a, Rcan1, Ripply3, Sh3bgr* and *Wrb*). We used the Transcription Element Search System program (TESS; see Methods) to identify potential TBEs in the first exons and/or promoter regions of those genes [26] (see Methods). Based on prior reports of possible effects on heart development, evolutionary conservation, the presence of TBEs and binding sites for other heart specific transcription factors, we selected *Adamts1* for further investigation*.*

**Fig. 3:**
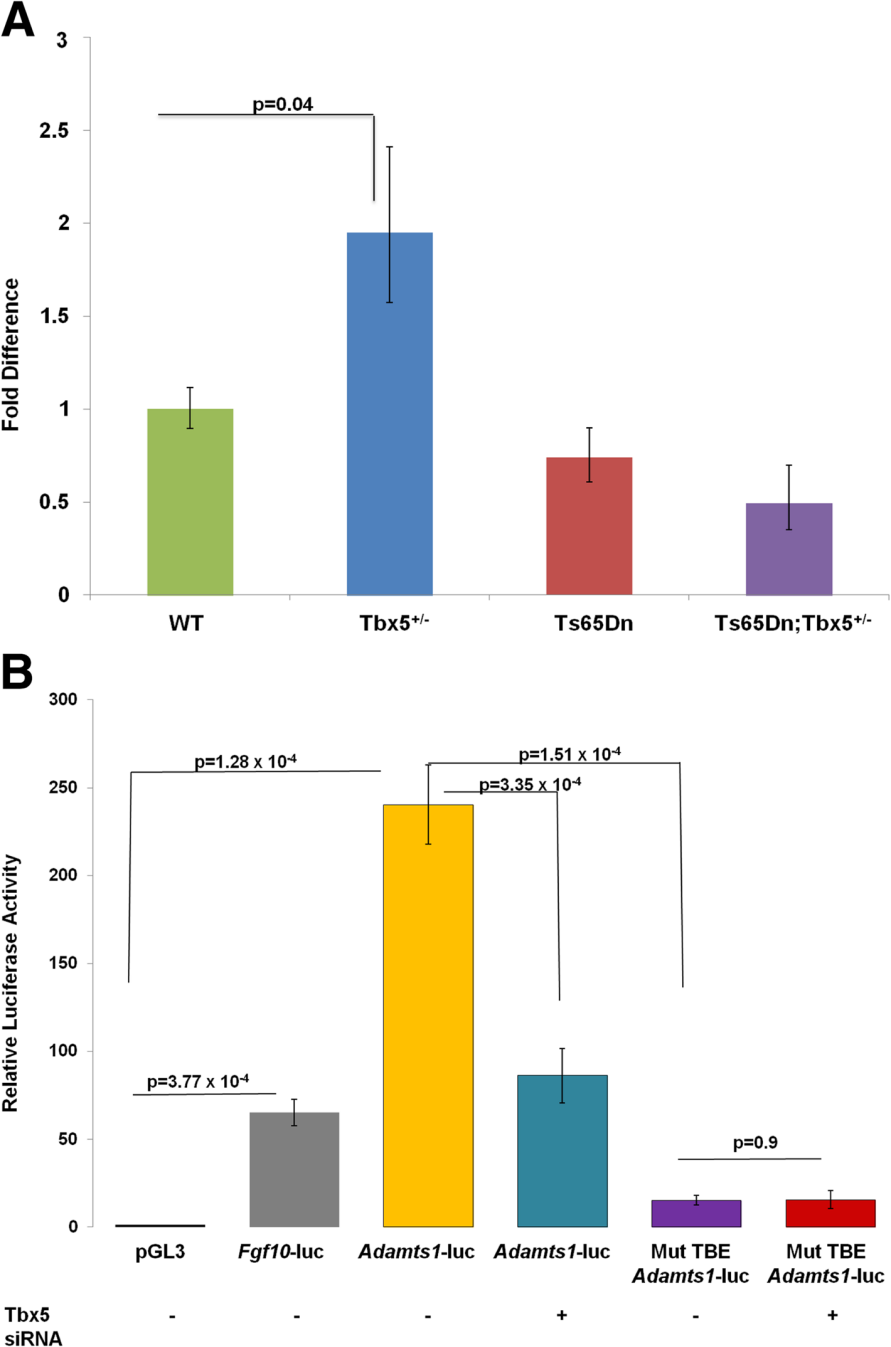
Interaction between *Tbx5* and trisomic gene, *Adamts1.* Quantitative PCR of *Adamts1* mRNA levels in pooled E11.5 hearts (**a**). *Adamts1* expression levels are significantly increased in euploid *Tbx5* ^*+/ -*^mice (1.95x, p = 0.04). No other changes were significant compared to the wild type expression. Error bars are standard deviation from the mean. TBX5 binding upstream of the *Adamts1* locus alters transcription (**b**). Cells transfected with the *Adamts1*-luc construct showed increased luciferase when compared to the pGL3 construct (p = 1.28 × 10^−4^). Cells transfected with the *Adamts*1-luc construct and *Tbx5* siRNA showed significantly lower luciferase levels when compared to those transfected with the *Adamts1*-luc construct alone (p = 3.35 × 10^−4^, compared to *Adamts*1-luc). Luciferase levels dropped significantly when the TBE was mutated to inhibit TBX5 binding (p = 1.51 × 10^−4^). Addition of *Tbx5* siRNA did not affect transcription when the TBE was mutated (p = 0.9) Error bars are standard deviation. The region inserted into the basic pGL3 vector is 121 bp upstream of the *Adamts1* gene, contains a putative TBE and associated with TBX5 in a ChIP study. *Tbx5* over expressing cells were harvested 48 h after transfection. The FGF10-luciferase construct was used as a positive control. Graph represents one experiment representative of all replicates

*Adamts1* encodes an extracellular matrix protease. Transcript levels were increased in *Tbx5*^*+/−*^ mice (p = 0.04) (Fig. 3a), suggesting that *Tbx5* may act as a repressor of *Adamts1*. Using TESS, we found a putative TBE upstream of the *Adamts1* locus and tested it using a luciferase reporter assay. The putative site, located 229 bp upstream of the *Adamts1* transcription start site, was identical to the canonical TBE (RGGTGTVR) [27]. He *et al.* [25] found that this region is bound by TBX5 in a chromatin immunoprecipitation assay. A 238 bp region containing the putative site and located 121 bp upstream of the *Adamts1* transcription start site, was amplified by PCR and cloned into the pGL3 luciferase vector (Additional file 6: Figure S2).

We transfected the construct into cells stably over expressing *Tbx5* (Additional file 7: Figure S3). Luciferase levels were increased in cells transfected with the *Adamts1*-luc construct compared to cells transfected with empty pGL3 vector (p = 1.28 × 10^−4^) (Fig. 3b). Introduction of siRNA directed at *Tbx5* reduced this effect (p = 3.35 × 10^−4^). The putative TBE was mutated to inhibit TBX5 binding and luciferase levels decreased compared to the construct with the intact TBE (p = 1.51 × 10^−4^).

*Tbx5* is generally considered to be an inducer of transcription, consistent with the increase in luciferase signal when TBX5 binds the upstream *Adamts*1 region included in the construct. However, Adamts1 expression is increased when Tbx5 is reduced in *Tbx5*^*+/−*^ mice (Fig. 3a). This discrepancy is likely due to the involvement of distal elements in transcriptional regulation that were not included in the construct and/or the lack of critical co-regulators in 3T3 cells. It is not surprising that *in vivo* results would differ in the presence of the entire promoter, as well as other transcription factors and co-regulators.

### Atrial isomerism in Ts65Dn;*Tbx5*^*+/−*^ mice

**Fig. 4:**
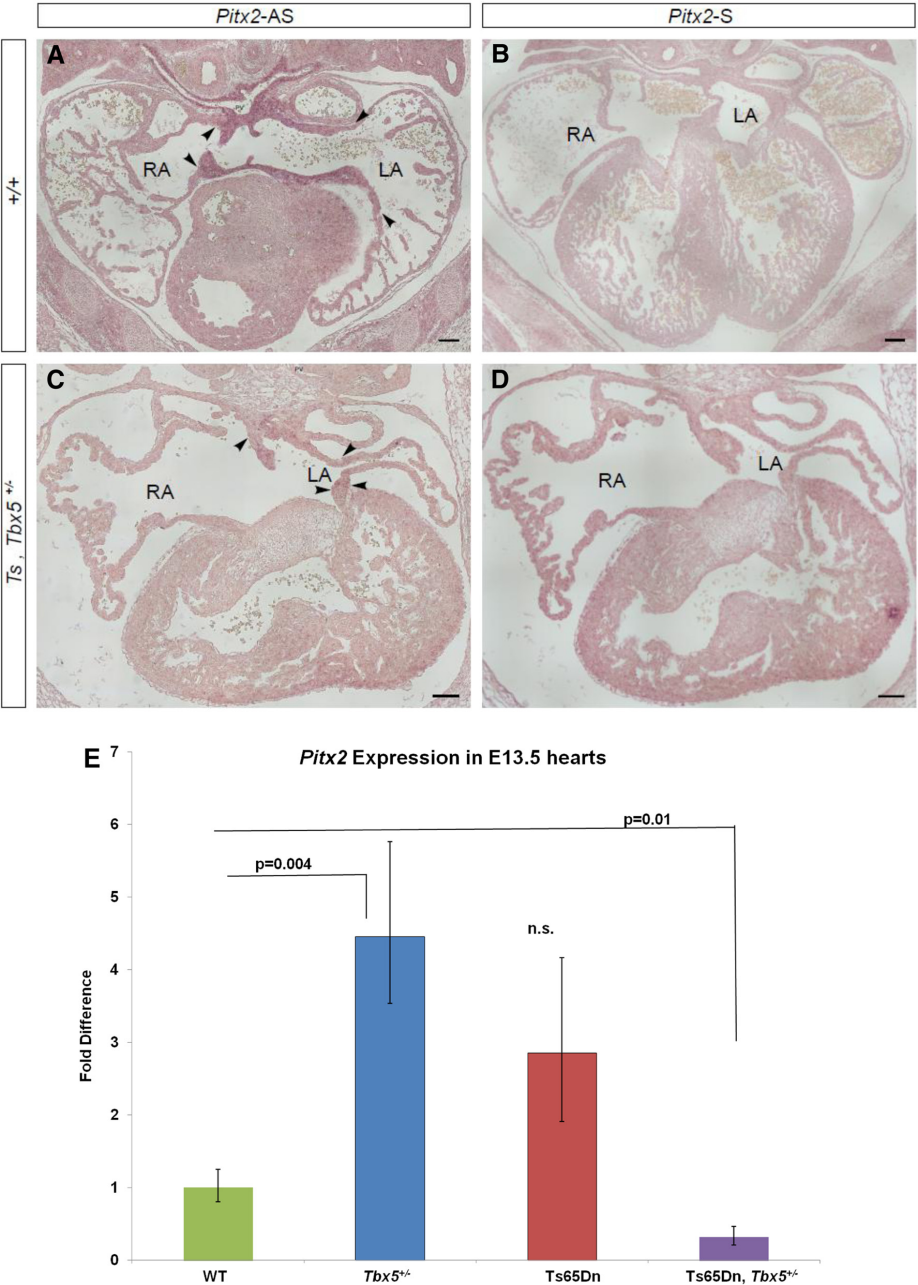
*Pitx2* expression in E13.5 WT and trisomic, *Tbx5* ^+/−^ embryos. *In situ* hybridization of coronal sections of wild type (**a**,**b**) and Ts65Dn;*Tbx5* ^+/−^ (**c**,**d**) mice. *Pitx2* expression can be seen in the left atrium of the WT animals (**a**). *Pitx2* expression is much weaker in the trisomic, *Tbx5* ^+/−^ mutants (**c**). Scale bar equals to 100 μm. RA/LA: right/left atrium. *In situ* hybridization results were verified through quantitative PCR in E13.5 hearts (**e**). Error bars are standard deviation from the mean

Overriding aorta and Gerbode’s defect are seen more often in Ts65Dn;*Tbx5*^*+/−*^ mice than in their *Tbx5*^*+/−*^ littermates or euploid controls. We hypothesized that these might be accompanied by atrial isomerism. In this situation both atria are mirror images of one another and lack the typical morphological and molecular characteristics of either the right or left atrium. Normally, *Pitx2* is specifically expressed in the left atrium and it is absent or ectopically expressed in the right atrium in cases of atrial isomerism [28]. To determine whether atrial isomerism occurred in Ts65Dn;*Tbx5*^*+/−*^ mice, *in situ* hybridization for *Pitx2* was performed on coronal paraffin sections of E13.5 thoraxes. *Pitx2* expression can be seen in the left atrium of the heart in euploid embryos, but that expression is much weaker in the mutants (Fig. 4a,c). These results were confirmed by quantitative PCR of whole hearts from E13.5 embryos (Fig. 4e), where *Pitx2* expression was reduced in Ts65Dn;*Tbx5*^*+/−*^ compared to euploid littermates (p = 0.01). The expected expression pattern was observed in other organs (Additional file 8: Figure S4). The weak *Pitx2* expression in the atria of Ts65Dn,*Tbx5*^*+/*^ embryos suggests that these embryos have defects in laterality (i.e., atrial isomerism). Thus, atrial isomerism likely contributes to the significant increase in overriding aorta in the Ts65Dn;*Tbx5*^+/−^ animals.

## Discussion

The interaction between *Tbx5* dosage and trisomy resulted in a significant increase in defects of aortic alignment and revealed a potential effect on left-right patterning of the heart*.* The action of genetic modifiers in heart development is evident from the differences in penetrance and patterns of heart phenotypes in DS, in DS mouse models and when the *Tbx5* null allele is bred onto different mouse genetic backgrounds. In addition, Holt-Oram patients who inherit the same *TBX5* mutation have variable heart phenotypes, indicating that additional factors affect development [29]. Previous work from our lab and others has shown how interactions between trisomy and disomic modifier genes, both in humans and in mouse models, can adversely affect heart development [8, 9]. For example, VEGF-A, HEY2 and the matricellular protein CRELD1 each have an impact, but they represent a small subset of genes in which variants contribute risk of CHD. Individuals with DS and mutations in *TBX5* displaying OA and a number of additional defects have been described [30]. Isolated OA is not frequent in DS, however it is a component of Tetralogy of Fallot which occurs in 1–8 % of DS births, far higher than in the population at large [2]. Our results demonstrate an interaction between altered *Tbx5* expression and trisomy with increased OA as a primary outcome.

Disruptions in the left-right patterning pathway frequently lead to the kinds of heart defects seen here. OA is considered to be a milder form of double outlet right ventricle (DORV), the most common defect seen in *Pitx2* knockout mice [31], arising in part due to aortic shifting [32]. Reducing the level of *Pitx2* expression is sufficient to cause septal and valve defects [33]. Mice lacking the *Pitx2* and *Cited2* genes which are involved in left-right patterning exhibit phenotypes similar to the ones reported, including Gerbode’s defect and overriding aorta [32–37]. *Pitx2* is an important determinant of left-right asymmetry of the heart and gut [28, 33, 37–40] and confers left atrial identity; its misexpression can result in atrial isomerism [28, 39, 41]. T-box family transcription factors are known to regulate *Pitx2* expression [42], and *Pitx2* regulates *Tbx5* expression in the abdominal wall during development [43]. Ts65Dn;*Tbx5*^*+/−*^ embryos have reduced *Pitx2* expression, which can contribute to atrial isomerism and aortic defects.

The left-right signaling pathway is affected when trisomy and *Tbx5* haploinsufficiency are combined. We suggested previously that a universal response deficit to Sonic hedgehog signaling due to trisomy could explain many of the clinical features of DS [44]; hedgehog signaling plays a regulatory role in left-right patterning and can regulate *Pitx2* expression [45–47]. Expression of Sonic hedgehog in the second heart field is essential for proper growth of the dorsal mesenchymal protrusion, without which AVSD occurs [48]. A response deficit to SHH in Ts65Dn could contribute to the altered localization of *Pitx2* expression seen here.

Finally, we discovered that mice with reduced *Tbx5* dosage show increased expression of the *Adamts1* gene. *Adamts1* is important in the development of the extracellular matrix in endocardial cushions [49], and is a regulator of the VEGF pathway [50]. It is interesting that individuals with DS who have an AVSD show an excess of deleterious variants in VEGF pathway genes relative to those with DS who do not have CHD [8]. Reduced *Tbx5* dosage may contribute to the risk of heart defects by a role in transcriptional regulation of the *Adamts1* gene, a regulator of the VEGF pathway.

Defects in left-right patterning likely contribute to the significant increase in overriding aorta in Ts65Dn;*Tbx5*^*+/−*^ mice relative to either genetic defects in isolation. The mechanism likely involves reduced expression of *Pitx2*, a gene with known roles in laterality. In addition, we demonstrated that a change in *Tbx5* dosage affects transcription of *Adamts1*, suggesting it is a target of TBX5 in normal heart development *in vivo*.

## Conclusions

We have shown that *Tbx5* dosage and trisomy interact to affect the outcome of heart development, potentially altering left-right patterning of the heart. We provided evidence of weak *Pitx2* expression, important for left-right patterning, in the Ts65Dn;*Tbx5*^*+/−*^ mice*.* We have also provided evidence that TBX5 plays a role in regulation of the trisomic *Adamts1* gene. A valid TBX5 binding site was shown upstream of the *Adamts1* locus and mutation of the site significantly affected transcription.

## Methods

### Animal husbandry

Animals were maintained in a virus and antibody-free facility with food and water *ad libitum*. Ts65Dn mice (B6EiC3H-a/A-Ts(17^16^)65Dn) were obtained from The Jackson Laboratory and maintained as an advanced intercross on the B6;C3H background. *Tbx5* heterozygous null mice (*Tbx5*^+/−^) [20] were kindly provided by Dr. Jonathan Seidman. We backcrossed the *Tbx5*^*+/−*^ mice onto a C57BL/6J background for at least five generations. All data was taken after mice had been backcrossed for at least five generations. Progeny in Tables 1 and 2 were the result of separate experiments. Progeny in Table 1 were obtained by crossing *Tbx5*^*+/−*^ mice on a B6 background with wild type B6 mice (obtained from Jackson Laboratory) or B6;C3H mice bred in our lab. Progeny in Tables 2 and 3 were obtained at P0 via crosses between Ts65Dn females (B6;C3H) and *Tbx5*^*+/−*^ males on a B6 background. All procedures were approved by the Institutional Animal Care and Use Committee.

### Genotyping

We extracted genomic DNA from tail tips of mice by heating at 90 °C for 2 h in 10 mM NaOH, 0.2 mM EDTA and used for genotyping by PCR. Sequences of primers for Ts65Dn genotyping are as follows: C17F: 5′-GTGGCAAGAGACTCAAATTCAAC-3′; C16R: 5′-TGGCTTATTATTATCAGGGCATTT-3′; IMR5: 5′-AAAGTCGCTCTGAGTTGTTAT-3′; IMR6: 5′-GGAGCGGGAGAAATGGATATG-3′. For Ts65Dn genotyping, PCR was done under the following cycling conditions: 95 °C 3 min, (94 °C 10s, 58.7 °C 20s, 72 °C 27 s) for 31 cycles, 72 °C 5 min. For *Tbx5* genotyping, three primers designed to amplify either the wild type or null alleles were added together in each reaction [20]. The sequences of the primers for *Tbx5* genotyping are as follows: 3 F2: 5′-CCCAGCGGCAGGCGTAGAC -3′; Loxp-F: 5′-GCAGCGCAGTCCTCACCAG -3′; Loxp-R: 5′-AAATTCCAACCCCTTCCACAGAT -3′. The PCR was done under the following cycling conditions: 94 °C 3 min, (94 °C 30s, 59.7 °C 30s, 72 °C 1 min) for 35 cycles, 72 °C 10 min.

### Histology

We collected the progeny of Ts65Dn x *Tbx5*^*+/−*^ crosses at postnatal day 0 (P0) within hours of birth. We euthanized pups and removed and fixed thoraxes in 10 % formalin for at least 48 h. Tissues were embedded in paraffin, sectioned at 7 μm and stained with hematoxylin/eosin using standard methods. The heart morphology in each animal was scored under a dissecting stereomicroscope (Nikon SMZ1500, Japan) by at least two individuals blinded to genotype. Pictures were taken using the NIS-Elements Br software (Nikon, Japan).

Virtual Histology™ was performed on six Ts65Dn;*Tbx5*^*+/−*^ mice (Numira Biosciences, Salt Lake City, UT). Virtual Histology™ is a micro-CT based method that allows for high resolution imaging. Specimens were stained in a proprietary solution before being scanned at 20 μm resolution with a volumetric micro-CT instrument. Using this method tissues are left intact and imaging analysis can be used to create “virtual” histological sections.

### Identification of candidate genes

We examined 109 genes that are trisomic in Ts65Dn mice for localization of expression in the heart using the following databases: EMAGE gene expression database [51], VisiGene image browser [52], the Chromosome 21 gene expression atlas [53], and the MGI gene expression database [54]. Trisomic genes expressed in heart during development, according to the above databases, were compared to a list of genes bound by TBX5 in a chromatin immunoprecipitation (ChIP) study [25]. We generated a list of trisomic candidate genes expressed in heart and bound by TBX5 (Additional file 5: Figure S1, Additional file 9: Table S2). From the list of 43 genes, we chose 6 as top candidates. The top 6 genes were the only genes out of the 43 which had a TBE in their promoter regions, were bound by other heart specific transcription factors, had high evolutionary conservation and were previously implicated in heart development. The Transcription Element Search System [26] was used to search for the TBE (RGGTGTVR) in regions of the 43 candidate genes that had a known or speculated role in heart development [27].

### Real-time analysis of gene expression

We extracted total RNA from the hearts of embryos using the RNeasy mini kit (Qiagen, Venlo, Netherlands). cDNA synthesis was carried out with the First-Strand cDNA synthesis kit (Life Sciences Advanced Technologies, St. Petersburg, FL) using 1 μg of total RNA as template. PCR was carried out on a 7500 Real-Time PCR System (Applied Biosystems, Carlsbad, CA). *Tbx5* was quantified using a Taqman FAM-labeled pre-designed assay (Mm0195728_m1) from Applied Biosystems and normalized to a *Gapdh* VIC-labeled assay (Mm99999915_g1) using Taqman Gene Expression master mix. POWER SybrGreen master mix was used for all other expression assays (Applied Biosystems, Carlsbad, CA). Primers used are as follows: *Adamts1*-F: 5′-CACGTGTGACACTCTCGGAA-3′; *Adamts1*-R: 5′-CGTGCGGCATGTTAAACACA-3′; *Gapdh*-F: 5′-TGCACCACCAACTGCTTAG-3′; *Gapdh*-R: 5′-GATGCAGGGATGATGTTC-3′; *Pitx2*-F: 5′-GCAGCCGTTGAATGTCTCTTC-3′; *Pitx2*-R: 5′-GTCCGTGAACTCGACCTTTTT-3′. PCR was done under the following cycling conditions: 95 °C 15 min, (95 °C 15 s, 60 °C 1 min) for 40 cycles, followed by a melt curve analysis between 95 and 60 °C. Expression of candidate genes was normalized to *Gapdh.* Fold change expression values were determined using the delta delta Ct method [55].

### Creation of *Tbx5* constitutively over-expressing cells

We obtained a *Tbx5* cDNA clone from the I.M.A.G.E. consortium and subcloned it into the pcDNA3.1+ vector (Life Technologies, Carlsbad, CA), putting it under the control of the CMV promoter. NIH-3T3 cells were transfected with the pcDNA-Tbx5 construct using Lipofectamine 2000 (Life Technologies, Carlsbad, CA). Stably transfected cell lines were selected with 1 mg/ml Geneticin (Gibco, Carlsbad, CA). We maintained the cells in Dulbecco’s Modified Eagle Medium with high glucose (4.5 g/L) (Life Technologies, Carlsbad, CA), 10 % fetal bovine serum, 1 mg/ml Geneticin, 1X glutamine and penicillin/streptomycin at 37 °C and 5 % CO_2_.

### Functional studies and luciferase assay

We amplified the portion of the promoter region of *Adamts1* that was bound by TBX5 in a ChIP study [25], by PCR with the insertion of cut sites for KpnI and XhoI. PCR primers are as follows, F: 5′-GGCGCTTATGGTACCTGGTCACACTTTTTTTGG-3′; R: 5′- GGCGCTTATCTCGAGCACCTTCACAGAGGCTCA-3′. The amplified region was subcloned into the pGL3 basic vector (Promega, Madison, WI). Site directed mutagenesis was used to mutate the putative TBE site found upstream of the *Adamts1* promoter. The following primers were used to mutate the putative TBE site and insert an EcoRI restriction site, Primer 1: 5′-CACAGCTCGTCACTCTGGGAATTCAAGACGCCGAAACAGCGCTG-3′; Primer 2: 5′-CAGCGCTGTTTCGGCGTCTTGAATTCCCAGAGTGACGAGCTGTG-3′. We transfected the constructs into NIH-3T3 cells constitutively over expressing *Tbx5* (Additional file 7: Figure S3). *Tbx5* siRNA and scrambled siRNA were purchased from Life Technologies (Carlsbad,CA). Luciferase levels were measured in a 1450 Wallac Jet MicroBeta liquid scintillation and luminescence counter (Perkin-Elmer, Waltham, MA) using the Dual Luciferase Assay Kit (Promega, Madison, WI).

### *In Situ* hybridization

E13.5 embryos were fixed in 4 % paraformaldehyde for 1 h, rinsed in 1X PBS three times for 5 min and then dehydrated in 25, 50 and 70 % ethanol for 1 h each. After dehydration the embryos were stored at −20 °C in 70 % ethanol until embedded in paraffin and sectioned according to standard methods. We performed *in situ* hybridization using traditional dioxigenin (DIG)-labeled RNA probes detected with alkaline phoshatase (AP)-conjugated anti-dioxigenin antibody using nitro blue tetrazolium chloride / 5-bromo-4-chloro-3-indolyl-phosphate toluidine-salt (NBT/BCIP) substrate. DIG RNA labeling mix, anti-DIG-AP antibody, blocking reagent, NBT/BCIP were all purchased from Roche Applied Science (Indianapolis, IN) and used according to the manufacturer’s instructions with minimal modifications. The *Pitx2* antisense and sense probes were described earlier [56]. Images were taken with the Leica DMRB upright light microscope using the Leica Application Suite Software v4.3 (Leica Microsystems-W. Nuhsbaum Inc., McHenry, IL). Composite images were compiled using Adobe Illustrator CS4 (Adobe Systems, San Jose, CA).

### Statistical analysis

The incidence of various heart defects for different genotypes was compared by Fisher’s test using GraphPad Prism version 5. A 2×2 contingency table was made comparing the two groups for each separate phenotype. The relative quantification of gene expression from different genotypes was compared by the student’s *t*-test using delta Ct values. All tests were 2-tailed and P < 0.05 were considered significant.

## Additional files

Additional file 1:
**Video 1.** https://jh.box.com/s/z3o7797mxgmiw5owbxcrey78o69y2tir. VSD in a Ts65Dn; *Tbx5* ^*+/−*^ P0 animal. 3D rendering of the heart of a P0 Ts65Dn; *Tbx5* ^*+/−*^ depicting a VSD. At the start of the video a coronal view of the heart can be seen. The left and right atria as well as the ventricles are shown. The heart then rotates so the view is now transverse. In the transverse view the heart is slowly cut away revealing the interior ventricles and a ventricular septal defect. Additional file 2:
**Video 2.** https://jh.box.com/s/3peeo5dazhdkil23ffxb62xblloybsnk. ASD in a Ts65Dn; *Tbx5* ^*+/−*^ P0 animal. 3D rendering of the heart of a P0 Ts65Dn; *Tbx5* ^*+/−*^ depicting an ASD. At the start of the video a coronal view of the heart can be seen. The left and right atria as well as the ventricles are shown. The heart rotates to a sagittal view. The heart is then slowly cut away revealing the interior of the right atria and an atrial septal defect. Additional file 3:
**Video 3.** https://jh.box.com/s/2loh3n0x55hbmstcu4kxn9rxt3tmf2x6. AVSD in a Ts65Dn; *Tbx5* ^*+/−*^ P0 animal. CT scan of a P0 Ts65Dn; *Tbx5* ^*+/−*^ depicting an AVSD. The video gives a coronal view of data collected during a micro-CT scan. The first look at the heart can be seen at 00:05. The video cuts away to reveal an atrioventricular septal defect in the heart, most clearly seen at 00:16. Additional file 4: Figure S1. Identifying trisomic gene candidates for *Tbx5* interaction. Gene expression databases were searched to find expression domains of all Ts65Dn trisomic genes. Genes that were expressed in the heart during development were further investigated for interaction with TBX5 in a ChIP study. Additional file 5: Figure S2. Region of *Adamts1* gene inserted into pGL3 vector. The 238 bp region highlighted in gray contains the canonical T-box binding site (RGGTGTVR) and was amplified by PCR and cloned into a pGL3 luciferase vector (Promega). This region was associated with TBX5 in a ChIP study. The TBE is located 229 bp upstream of the *Adamts1* transcription start site. Additional file 6: Figure S3.
*Tbx5* mRNA expression in transfected cells. Quantitative PCR was used to measure *Tbx5* expression in NIH-3T3 cells transfected with a *Tbx5-*pcDNA3.1+ construct using Lipofectamine 2000 (Life Technologies, Carlsbad, CA). Stably transfected cells expressed about 70 times as much *Tbx5* as the NIH-3T3 cells. Stably transfected cell lines were selected with 1 mg/ml Geneticin (Gibco, Carlsbad, CA). Additional file 7: Figure S4. Expression of *Pitx2* with *in situ* hybridization (ISH) in E13.5 WT and trisomic, *Tbx5* ^+/−^ mutants in other areas as pituitary (A, E, I, M), tooth bud (B, F, J, N), and midbrain(C, G, K, O) in sagittal sections and spinal cord in horizontal sections (D, H, L, P). Embryo faces to the right in sagittal sections. Sections come from same animals as used for the heart studies and processed for ISH at the same time. Spinal cord photos are from the exact same slides photographed for heart studies. Arrowheads in Panel D and E mark expected *Pitx2* staining in spinal cord neurons. *Pitx2*-AS: *Pitx2* antisense probe, *Pitx2S*: *Pitx2* sense probe. Scale bar equals to 100 μm. Additional file 8: Table S1. Number of animals with multiple defects Additional file 9: Table S2. Forty-three Ts65Dn trisomic genes expressed in heart during development and bound by TBX5 in a ChIP experiment.

## Abbreviations

DS: Down syndrome
CHD: Congenital heart defect
Hsa21: Human chromosome 21
AVSD: Atrioventricular septal defect
ASD: Atrial septal defect
VSD: Ventricular septal defect
LV: Left ventricle
RV: Right ventricle
Mmu16: Mouse chromosome 16
Mmu10: Mouse chromosome 10
Mmu17: Mouse chromosome 17
OA: Overriding aorta
ChIP: Chromatin immunoprecipitation
TBE: T-box binding element
