# Supplementary figures and images for: The pattern of congenital heart defects arising from reduced Tbx5 expression is altered in a Down syndrome mouse model
Source: BMC Dev Biol. 2015 Jul 25;15:30. doi: 10.1186/s12861-015-0080-y (PMC4514943; doi:10.1186/s12861-015-0080-y)

## Slide 1
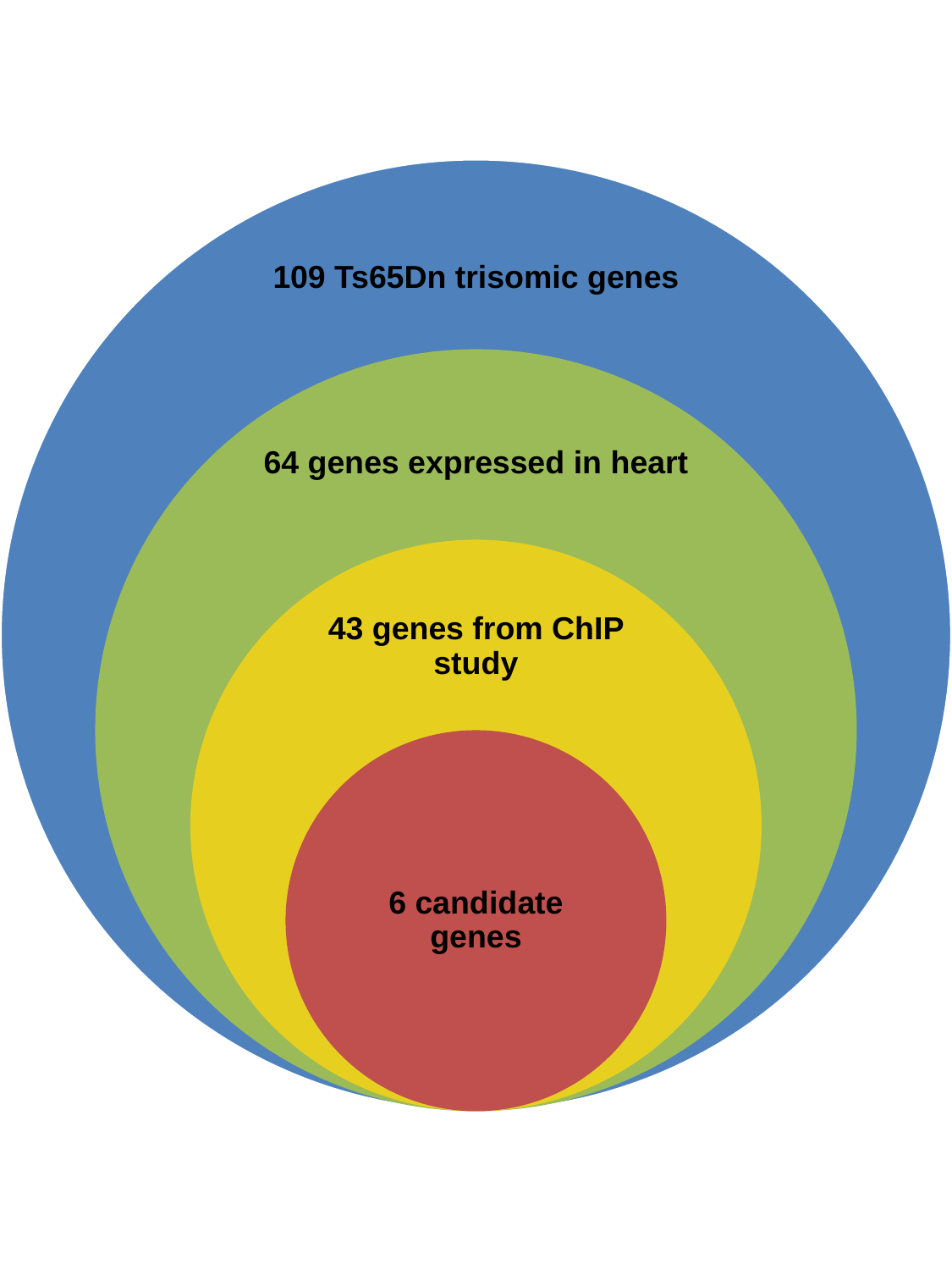

Supplement: Additional file 4: Figure S1. — Identifying trisomic gene candidates for Tbx5 interaction. Gene expression databases were searched to find expression domains of all Ts65Dn trisomic genes. Genes that were expressed in the heart during development were further investigated for interaction with TBX5 in a ChIP study. [file 12861_2015_80_MOESM4_ESM.pptx]

## Slide 1
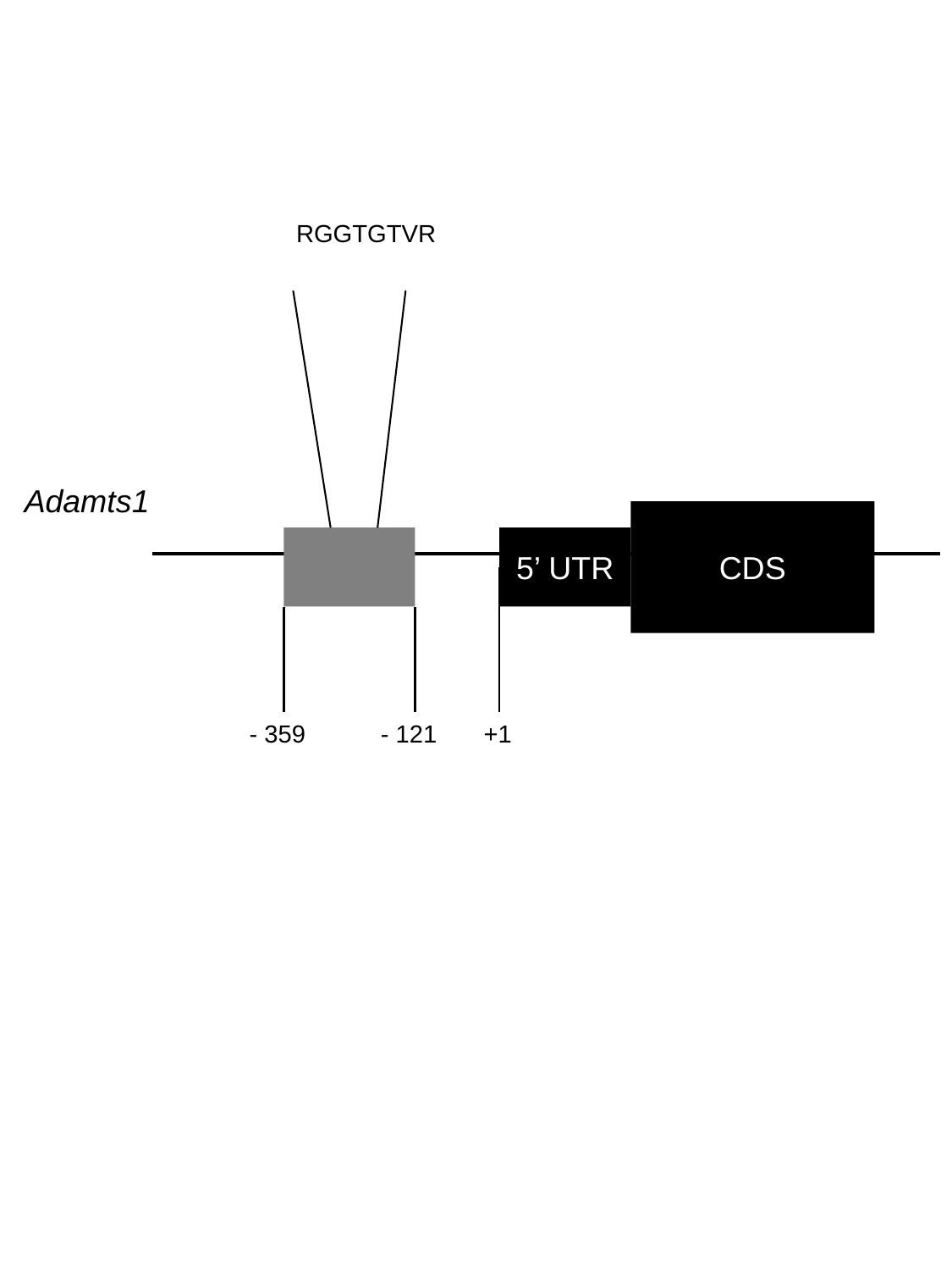

RGGTGTVR
Adamts1
CDS
5’ UTR
- 359
- 121
+1

Supplement: Additional file 5: Figure S2. — Region of Adamts1 gene inserted into pGL3 vector. The 238 bp region highlighted in gray contains the canonical T-box binding site (RGGTGTVR) and was amplified by PCR and cloned into a pGL3 luciferase vector (Promega). This region was associated with TBX5 in a ChIP study. The TBE is located 229 bp upstream of the Adamts1 transcription start site. [file 12861_2015_80_MOESM5_ESM.pptx]

## Slide 1
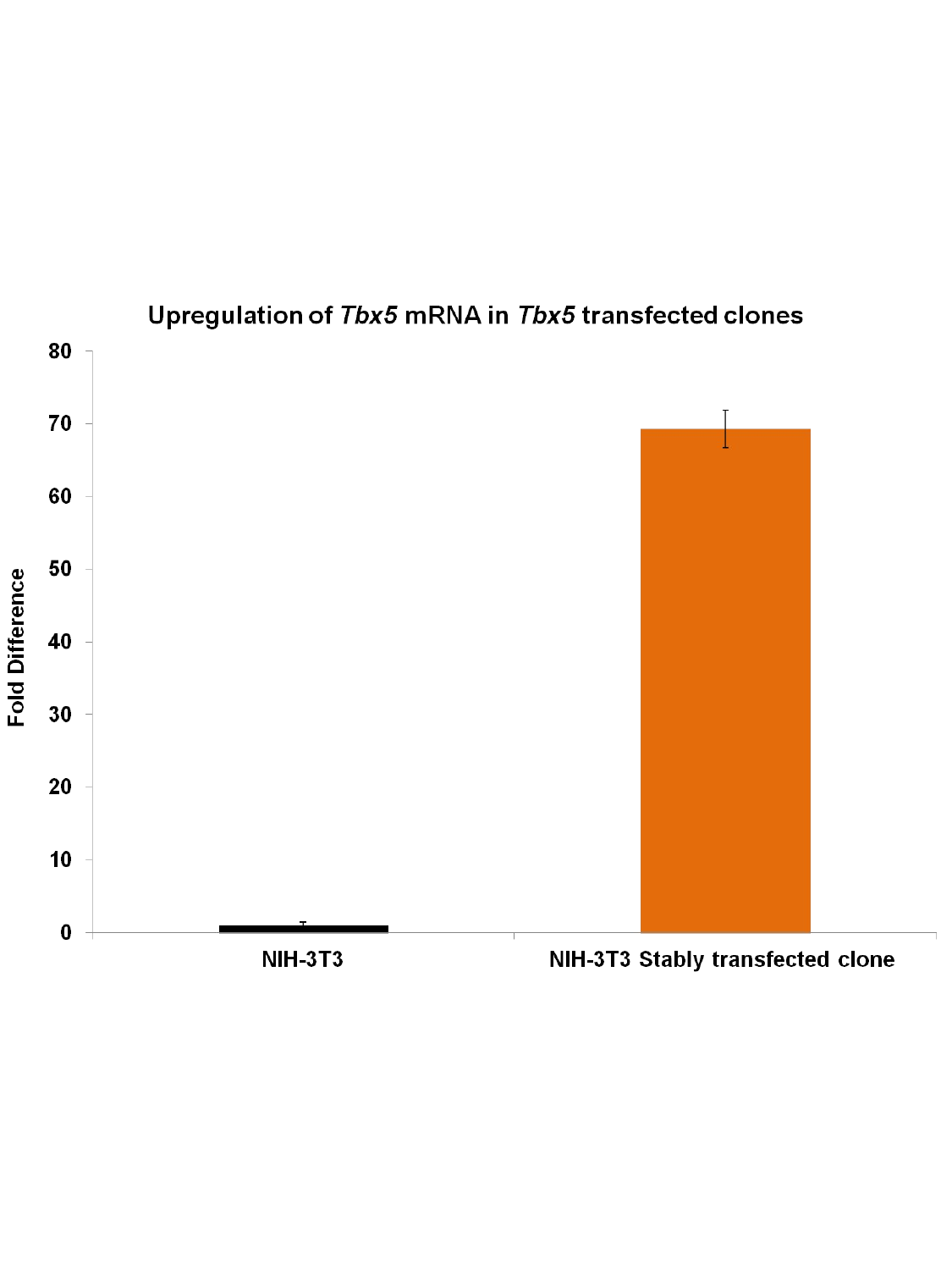

Supplement: Additional file 6: Figure S3. — Tbx5 mRNA expression in transfected cells. Quantitative PCR was used to measure Tbx5 expression in NIH-3T3 cells transfected with a Tbx5-pcDNA3.1+ construct using Lipofectamine 2000 (Life Technologies, Carlsbad, CA). Stably transfected cells expressed about 70 times as much Tbx5 as the NIH-3T3 cells. Stably transfected cell lines were selected with 1 mg/ml Geneticin (Gibco, Carlsbad, CA). [file 12861_2015_80_MOESM6_ESM.pptx]

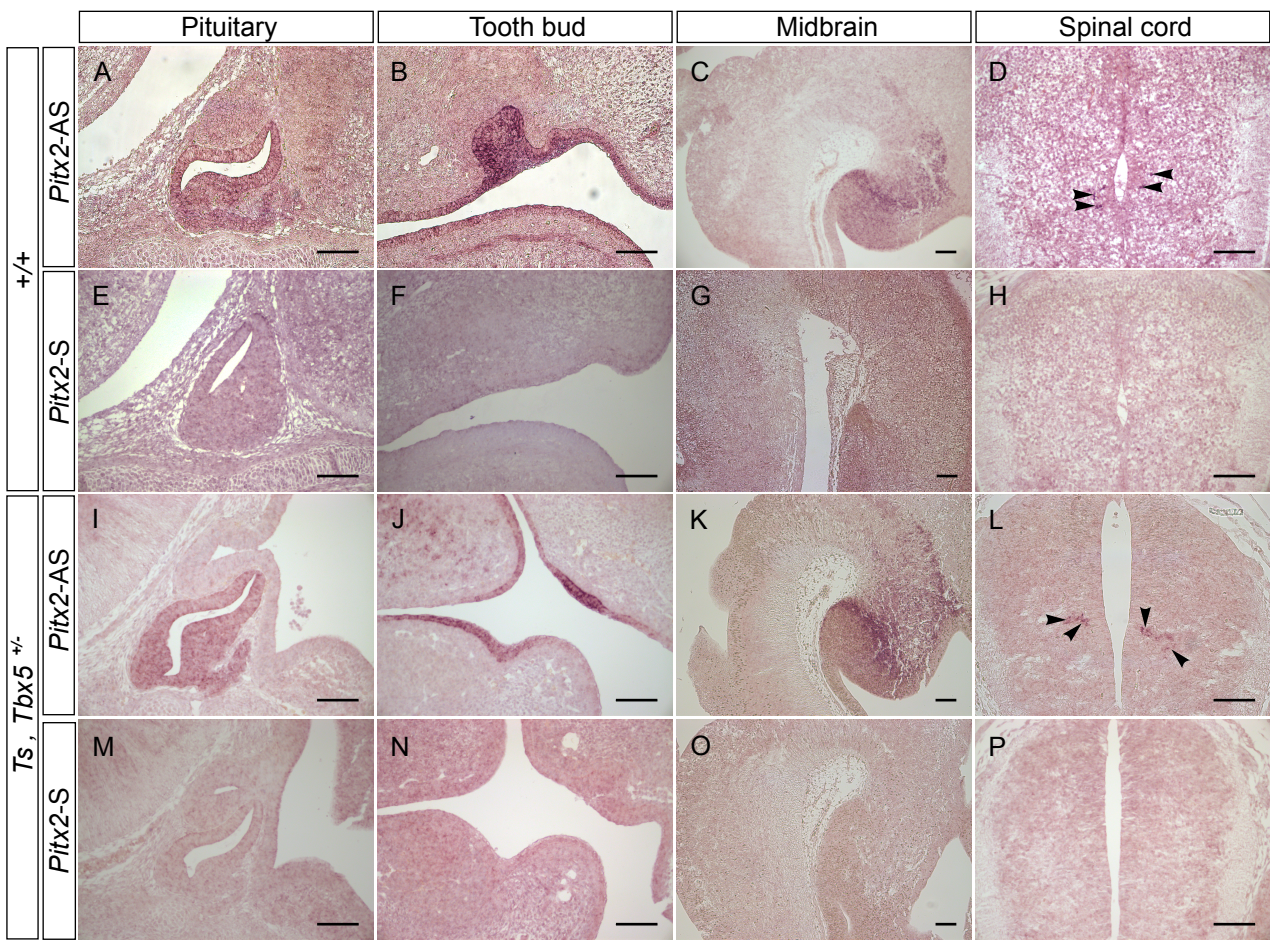

Supplement: Additional file 7: Figure S4. — Expression of Pitx2 with in situ hybridization (ISH) in E13.5 WT and trisomic, Tbx5 +/− mutants in other areas as pituitary (A, E, I, M), tooth bud (B, F, J, N), and midbrain(C, G, K, O) in sagittal sections and spinal cord in horizontal sections (D, H, L, P). Embryo faces to the right in sagittal sections. Sections come from same animals as used for the heart studies and processed for ISH at the same time. Spinal cord photos are from the exact same slides photographed for heart studies. Arrowheads in Panel D and E mark expected Pitx2 staining in spinal cord neurons. Pitx2-AS: Pitx2 antisense probe, Pitx2S: Pitx2 sense probe. Scale bar equals to 100 μm. [file 12861_2015_80_MOESM7_ESM.pdf]
