# Supplementary material for: The pattern of congenital heart defects arising from reduced Tbx5 expression is altered in a Down syndrome mouse model
Source: BMC Dev Biol. 2015 Jul 25;15:30. doi: 10.1186/s12861-015-0080-y (PMC4514943; doi:10.1186/s12861-015-0080-y)
Supplement: Additional file 8: Table S1. — Number of animals with multiple defects [file 12861_2015_80_MOESM8_ESM.doc]

**Supplemental Table 1. Number of animals with multiple defects**

| Genotype | No Defect | 1 defect | >1 defect | Total Mice |
| --- | --- | --- | --- | --- |
| *Tbx5*^+/-^ | 13 (32.5%) | 13 (32.5%) | 14 (35%) | 40 |
| Ts65Dn, *Tbx5^+/-^* | 5 (16.1%) | 6 (19.4%) | 20 (64.5%) | 31 |
