# Supplementary material for: The pattern of congenital heart defects arising from reduced Tbx5 expression is altered in a Down syndrome mouse model
Source: BMC Dev Biol. 2015 Jul 25;15:30. doi: 10.1186/s12861-015-0080-y (PMC4514943; doi:10.1186/s12861-015-0080-y)
Supplement: Additional file 9: Table S2. — Forty-three Ts65Dn trisomic genes expressed in heart during development and bound by TBX5 in a ChIP experiment. [file 12861_2015_80_MOESM9_ESM.doc]

**Supplemental Table 2.** Forty-three Ts65Dn trisomic genes expressed in heart during development and bound by TBX5 in a ChIP experiment.

| Ts65Dn genes bound by Tbx5 | | | |
| --- | --- | --- | --- |
| *Adamts1*  *Adamts5*  *App*  *Atp5j*  *Atp5o*  *Bace2*  *Bach1*  *Cbr1*  *Cbr3*  *Cryzl1*  *Donson* | *Dopey2*  *Dscr3*  *Dyrk1a*  *Erg*  *Ets2*  *Gabpa*  *Gart*  *Hmgn1*  *Hunk*  *Ifnar1*  *Ifnar2* | *Il10rb*  *Itsn1*  *Jam2*  *Kcne1*  *Morc3*  *Mrap*  *Mrpl39*  *Mrps6*  *Prdm15*  *Psmg1*  *Rcan1* | *Ripply3*  *Rwdd2b*  *Sh3bgr*  *Setd4*  *Sod1*  *Son*  *Tmem50b*  *Ttc3*  *Usp16*  *Wrb* |

^a^ He, A., et al., *Co-occupancy by multiple cardiac transcription factors identifies transcriptional enhancers active in heart.* Proc Natl Acad Sci U S A, 2011; **108**(14): 5632-7
